# Mydriatics in Refraction

**Published:** 1896-01

**Authors:** Arthur G. Bennett

**Affiliations:** 213 Franklin Street


### Correspondence.

### MYDRIATICS IN REFRACTION.

*To the Editor:*

SIR—The article by Dr. Satterlee in the December issue, on mydriatics in refraction, should not be allowed to pass without challenge. I believe it to be false in teaching, dangerous in practice and calculated to do an infinite amount of harm. The consensus of opinion of the leading ophthalmologists of the world is against him. This he practically admits when he says: "One or two French ophthalmologists and a few in this country are all that I call to mind who have discarded the routine use of mydriatics." I had it in my mind to quote him authorities in support of their use, but on making references I find such an embarrassment of riches that I refrain, and will only refer him to such writers as Donders the Great, Soelberg Wells, Brudenel Carter, Fuchs, Noyes,

Stevens, de Schweinitz, Jackson and in fact anybody of note who has written at all on refraction. The more modern and progressive the man, the more does he dwell on the necessity of refracting with the accommodation paralysed.

Taking Dr. Satterlee's objections in order:

1. The dilated pupil with a paralysed accommodation is an unnatural condition of the eye. The proof of this is the fact that the patient cannot afterward wear the glass accepted under the influence of the mydriatic.

*Answer.*—The dilated pupil is no more unnatural than is the condition that calls for the dilatation. If we were dealing with a normal organ no medication would be necessary; dilatation is part of the treatment. The same objection could be urged against distending a dilated stomach with $C.O._2$ in order to map out its abnormal dimensions, or against the dilatation of the sphincter ani to properly treat hemorrhoids. The proof offered is no proof at all; in the vast majority of cases the patient will wear with comfort a cylindrical lens accepted under a mydriatic, will always wear a minus lens, either simple, spherical, cylindrical or a combination of both, and in many instances will wear the full hyperopic correction, though it is deemed wiser in the majority of cases to make some allowance for the hypertrophy of the ciliary muscle and reduce the strength of the lens, but the most modern teaching is to give as nearly as possible a full correction.

2. The cornea and lens are frequently perfect in curvature in the immediate vicinity of the normal pupil, while a short distance away they have a greater curvature in one meridian than in another. The dilated pupil would bring out these imperfections that the normal pupil would not.

*Answer.*—The second part of this question answers the first. In a badly-illuminated room the pupil is dilated more or less, and in some individuals very widely, and particularly so in the class of patients most likely to suffer from eyestrain,—the anemic and the hysterical. Is a patient to suffer with asthenopia or sundry reflex ills because at the time of the examination, owing to the glare of the ophthalmometer disc, or the brilliant illumination of the ophthalmoscopic mirror, the pupil is contracted and no astigmatism is manifest?

3. Many people are weeks recovering from the mydriatics.

*Answer.*—I do not agree with Dr. Satterlee in his choice of an adjective. He would have been nearer the mark had he said "a few." Shall we deny the many the inestimable advantage of a correct diagnosis because a few people are inconvenienced for a few days? We might with equal reason eliminate opium from the materia medica because of some few people's idiosyncracies. In an experience of some thousand cases in private practice, and several thousand in infirmary work, I have yet to see a case in which the effect of the mydriatic (homatropine) lasted more than three days at the outside, and the vast majority had recovered in thirty-six hours.

4. In some cases of glaucoma the increase in tension is slight, and by no means as easily detected as the average text-book would lead one to infer. Any mydriatic in these cases produces permanent injury to the sight.

*Answer.*—Glaucoma does not usually affect patients under the age of presbyopia. At this age, no mydriatic is necessary, as Nature has paralysed the accommodation for us. This great danger is, therefore, to a great extent obviated. Still there are some few cases which occur earlier, and it has been noted in quite young patients, but these cases are extremely rare, and if the ophthalmologist makes a routine practice of using the ophthalmoscope with every patient before using a mydriatic, I do not see how he can make such a blunder.

5. Hyperesthesia of the retina exists in many cases of anemia, hysteria and the like. A large pupil allows still more light to irritate the sensitive retina and greatly increase the discomfort to the patient.

*Answer.*—Hyperesthesia of the retina is due, in most cases, to an error of refraction, and the cure is a properly fitted pair of spectacles. If Dr. Satterlee will tell his patients to wear a pair of smoked glasses until the effect of the mydriatic has disappeared, he will find this discomfort reduced to a minimum.

6. In the majority of cases of refraction the mydriatic is unnecessary.

On this point I take direct issue with Dr. Satterlee. I learned to do my refractive work without a mydriatic, and have learned in the bitter school of experience that my work without a paralysed accommodation was not satisfactory. I am familiar with the ophthalmometer, and am aware that it is a valuable instrument, though more in the way of suggestion than as an instrument of precision. At its best, however, it only determines whether or not astigmatism is present, tells you nothing as to its variety, whether simple or compound, whether hyperopic, myopic or mixed; tells you nothing as to the presence or absence of hyperopia or myopia, but deals solely with the asymmetry of the cornea, and is worse than useless in lenticular astigmatism. I can recall case after case where the astigmatism as determined by this instrument was utterly at variance with result as shown by the test lenses, not only in amount but in direction of the axis. I have seen differences as great as three diopters and repeatedly as much as one diopter. I have had the pleasure of re-refracting cases that have been wearing glasses prescribed by ophthalmologists who do not believe in a mydriatic, and who trust to time (and lots of it), the ophthalmoscope and ophthalmometer in their refractive work, and I have been able with the help of a mydriatic to prescribe glasses that were at least comfortable, which is more than could be said of the spectacles that had been prescribed without its use. A few cases by way of illustration: E. J. V. was wearing — 1.25 D. sph., took under a mydriatic + 1.50 D. sph.; J. M. was wearing + .50 D. sph., took under a mydriatic + 1.50 D. sph. ⊃ + .50 cyl. axis 90°; A. L. H. was wearing + .75 D. sph., took under mydriatic + .50 D. sph. ⊃ + .75 cyl. axis 90°; Dr. G. H. was wearing — 4.00 D. sph. each, took under mydriatic R. E. — 4.00 D. sph. ⊃ — 1.00 cyl. axis 140°, L. E. — 3.00 D. sph. ⊃ — 1.50 D. cyl. axis 15°; Mrs. H. S. wearing + 1.00 D. sph., took under mydriatic — 1.50 D. sph. ⊃ + 3.00 D. cyl. axis 90°; and I could recite dozens of similar cases, but these are sufficient to prove to me that these cases were not properly fitted without a mydriatic, and also proves to me the necessity of using one.

We are not now limited to the use of atropine with its two weeks' siege of blurred vision. Homatropine, the effects of which disappear in thirty hours in most cases, and in some less, and never exceeds three days, is all that is necessary as a rule.

A properly fitting glass will, I agree, relieve asthenopia, but it can be more accurately adjusted when the patient's accommodation is paralysed temporarily. We should not take a retrograde step and place ourselves in the same plane as the "clockmaker" and the "oculo-optician," as we most certainly shall do if we give up the use of our beneficent mydriatics. I prefer to rank myself with the large army of expert ophthalmic surgeons of this country who do use a mydriatic for refractive purposes, rather than with the small band of heretics comprised of "one or two French ophthalmologists and a few in this country" who do not.

ARTHUR G. BENNETT, M. D.

213 Franklin Street.

